# Immune modulation as a consequence of SARS-CoV-2 infection

**DOI:** 10.3389/fimmu.2022.954391

**Published:** 2022-08-30

**Authors:** Metin Yusuf Gelmez, Fatma Betul Oktelik, Ilhan Tahrali, Vuslat Yilmaz, Umut Can Kucuksezer, Nilgun Akdeniz, Esin Aktas Cetin, Murat Kose, Cigdem Cinar, Fatma Savran Oguz, Sevgi Besisik, Kaya Koksalan, Ozkan Ozdemir, Naci Senkal, Ahmet Gul, Erdem Tuzun, Gunnur Deniz

**Affiliations:** ^1^ Department of Immunology, Aziz Sancar Institute of Experimental Medicine, Istanbul University, Istanbul, Turkey; ^2^ Institute of Graduate Studies in Health Science, Istanbul University, Istanbul, Turkey; ^3^ Department of Neuroscience, Aziz Sancar Institute of Experimental Medicine, Istanbul University, Istanbul, Turkey; ^4^ Department of Internal Medicine, Istanbul Faculty of Medicine, Istanbul University, Istanbul, Turkey; ^5^ Department of Medical Biology, Istanbul Faculty of Medicine, Istanbul University, Istanbul, Turkey; ^6^ Division of Hematology, Department of Internal Medicine, Istanbul Faculty of Medicine, Istanbul University, Istanbul, Turkey; ^7^ Istanbul Medical Faculty Hospital Blood Center, Istanbul Faculty of Medicine, Istanbul University, Istanbul, Turkey; ^8^ Laboratory of Molecular Tuberculosis Epidemiology, Aziz Sancar Institute of Experimental Medicine, Istanbul University, Istanbul, Turkey; ^9^ Genome Studies Program, Institute of Health Sciences, Acıbadem Mehmet Ali Aydınlar University, Istanbul, Turkey; ^10^ Division of Rheumatology, Department of Internal Medicine, Istanbul Faculty of Medicine, Istanbul University, Istanbul, Turkey

**Keywords:** SARS-CoV-2, COVID-19, T cells, B cells, ILCs, NK cells

## Abstract

Erroneous immune responses in COVID-19 could have detrimental effects, which makes investigation of immune network underlying COVID-19 pathogenesis a requisite. This study aimed to investigate COVID-19 related alterations within the frame of innate and adaptive immunity. Thirty-four patients clinically diagnosed with mild, moderate and severe COVID-19 disease were enrolled in this study. Decreased ILC1 and increased ILC2 subsets were detected in mild and moderate patients compared to healthy controls. NK cell subsets and cytotoxic capacity of NK cells were decreased in severe patients. Moreover, CD3^+^ T cells were reduced in severe patients and a negative correlation was found between CD3^+^ T cells and D-dimer levels. Likewise, moderate and severe patients showed diminished CD3^+^CD8^+^ T cells. Unlike T and NK cells, plasmablast and plasma cells were elevated in patients and IgG and IgA levels were particularly increased in severe patients. Severe patients also showed elevated serum levels of pro-inflammatory cytokines such as TNF-α, IL-6 and IL-8, reduced intracellular IFN-γ and increased intracellular IL-10 levels. Our findings emphasize that SARS-CoV-2 infection significantly alters immune responses and innate and acquired immunity are differentially modulated in line with the clinical severity of the disease. Elevation of IL-10 levels in NK cells and reduction of CD3^+^ and CD8^+^ T cells in severe patients might be considered as a protective response against the harmful effect of cytokine storm seen in COVID-19.

## Graphical Abstract

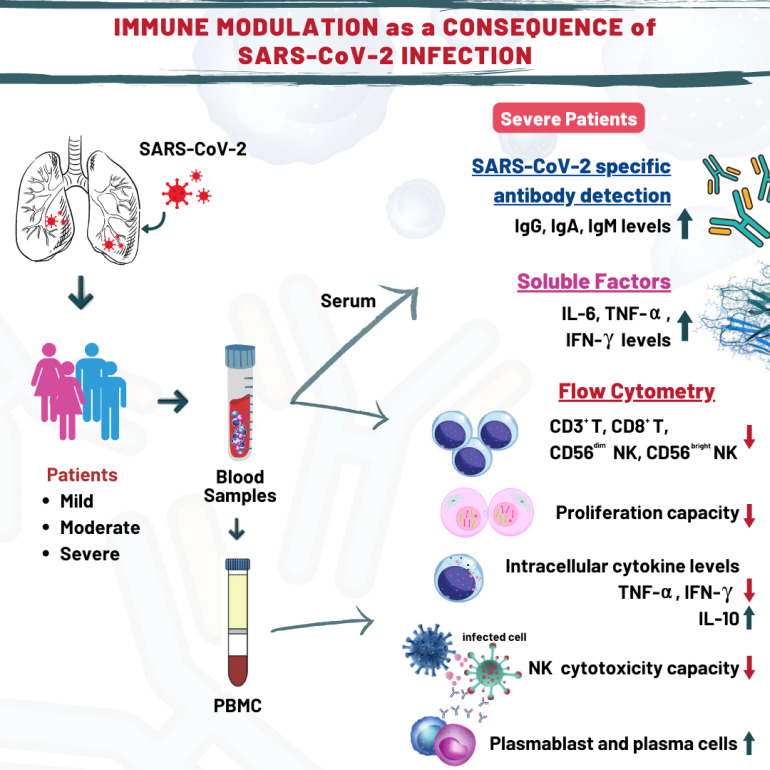

## Introduction

Severe acute respiratory syndrome coronavirus 2 (SARS-CoV-2) has been first identified in Wuhan, China at the end of 2019 and caused Coronavirus Disease (COVID)-19 which has a wide clinical spectrum from asymptomatic to mild symptoms, severe pneumonia, respiratory distress syndrome, or death (1–3). As of April 2022, it has been reported that there are approximately 15 million cases in Turkey and 492 million in the world, and more than 6 million deaths were seen all around the world.

In the immune defense against COVID-19, stimulation of antibodies and T-cell responses are particularly important because the coronavirus may induce “erroneous” immune responses that can lead to immunopathology and potential complications as consequences of infection. In many studies, both adaptive and innate arms of the immune response have been demonstrated to be effective in defense against SARS-CoV-2 infection (4–7).

Innate lymphoid cells (ILCs), including Natural Killer (NK) cells are the members of innate immunity derived from common lymphoid progenitors (8). ILCs have roles in inflammatory responses, tissue repair and neutrophil infiltration, and protective roles in respiratory infections and lung diseases (9–12). On the other hand, NK cells identified as CD3^−^ lymphocytes have been divided in two main subgroups, such that CD3^−^CD16^+^CD56^dim^ cells have a cytotoxic function and CD3^−^CD16^−/+^CD56^bright^ cells have great cytokine production capability (8, 13). NK cells have strong cytotoxic function against virus-infected cells and tumor cells. Considering the protective role of ILCs and NK cells in defense toward viral infections, it is important to determine their involvement in the pathogenesis of COVID-19.

B and T cells have a pivotal role in anti-viral immunity by secreting antibody, cytokines and cytotoxic enzymes, respectively (7). CD4^+^ T lymphocytes secrete various cytokines such as interferon (IFN)-γ and tumor necrosis factor (TNF)-α, which contribute to the differentiation of B lymphocytes into IgG- or IgA-secreting plasma cells and stimulate other immune cells (4).

It has been demonstrated that SARS-CoV-2-specific antibodies as well as memory B and T cell responses provide long-term protection to infection with SARS-CoV-2 (14). Antibodies against the spike (S) protein of SARS-CoV-2 are found after the onset of symptomatic responses (15). A subgroup of COVID-19 patients was revealed not to develop long-term antibodies against SARS-CoV-2 infection.

Most of COVID-19 patients worldwide are classified as mild or moderate cases (16). On the other hand, weak immune responses, older age and some chronic disorders like hypertension, diabetes, obesity and coronary artery disease were reported to be risk factors for severe course of COVID-19 (2, 17). The studies showed that lymphopenia and immune responses detected in COVID-19 patients were associated with disease progression (18–20). Several studies suggested that decreased CD4^+^ and CD8^+^ T, B or NK cell levels in severe patients compared to mild or moderate patients might provide a prognostic information about the disease (6, 21). Moreover, the levels of pro-inflammatory cytokines such as interleukin-6 (IL-6), IL-8 and TNF-α tended to be increased in severe COVID-19 patients, which could also be indicators of the disease progression (22).

There are differences in the treatment protocols applied against SARS-CoV-2 infection in the world. In Turkey, hydroxychloroquine and favipiravir was used in all patients until May 2021, and January 2022, respectively (23).

It is important to determine the competence of immune responses against SARS-CoV-2 for understanding the immunopathogenesis of COVID-19. Thus, this study aimed to determine the functionality of T, B, NK and ILC subgroups in order to evaluate the innate and acquired immune responses in mild, moderate and severe COVID-19 patients. With this study, the profile of immune responses against the infection with SARS-CoV-2 in Turkish population has been demonstrated in detail.

## Material and methods

### Study population

Thirty-four patients with COVID-19 [female (n=9) and male (n=25)] were included into the study from the Pandemy Clinic of Istanbul University, Istanbul Faculty of Medicine (Istanbul, Turkey). All patients were enrolled in the study from June to September 2020 when the first peak of the pandemic was observed in our country. All COVID-19 patients were selected according to positive SARS-CoV-2 test from a nasal swab based on RT-PCR, and have typical chest computed tomography (CT) findings.

Severity of illness was defined as follows: (1) Mild for patients: who do not require inpatient hospitalization; (2) Moderate for patients: moderate/severe pneumonia (bilateral infiltration and/or multiple mottling and ground-glass opacity), hypotension, dyspnea, mild pneumonia (unilateral infiltration) but having severe biochemical or hematological parameters such as ferritin value >1000 ng/mL or high-sensitivity C reactive protein (hsCRP) >40 mg/dL or lymphopenia; (3) Severe for patients: Hospitalized patients who require more than 10 liters per minute (LPM) of supplemental oxygen, or who require high flow oxygen, non-invasive ventilation, mechanical ventilation, extra-corporeal membrane oxygenation (ECMO), or with other forms of organ failure such as acute renal failure requiring renal replacement therapy or shock requiring vasopressors (24).

All patients were under treatment with hydroxychloroquine and azithromycin at the time of blood sampling. No patients were vaccinated against COVID-19. All moderate and severe patients were receiving favipiravir, and 5 of 10 severe patients were receiving tocilizumab treatment (25). None of the patients had a concomitant autoimmune or infectious disorder or received additional treatments.

Age and gender-matched healthy controls (n=10) who had no clinical history of cancer, autoimmunity, infection, immunosuppressive medication, or smoking were also included into the study. Peripheral blood samples were collected from patients and healthy controls. Written informed consent was obtained according to the Helsinki declaration and the study protocol was approved by the local Ethics Committee number: 11/05/2020-81367 of Istanbul Faculty of Medicine, Istanbul University.

### Flow cytometry analyses of lymphocyte subsets

Cell surface staining was processed using whole blood lysis method. Freshly drawn heparinized peripheral blood samples were labeled with monoclonal antibody (mAb) panel for T lymphocyte subsets [anti-CD3-AlexaFlour700, -CD4-APC, -CD8-APC/CY7, -CD45-BV510, -CD45RA-FITC, -CD45RO-PE, -CCR7-PE and -HLA-DR-BV711 (all from Biolegend, San Jose, CA, USA)], mAb panel for B lymphocyte subsets [anti-CD19-APC, -CD24-PE, -CD27-PE/CY7, -CD38-AlexaFlour700, -CD45-BV510, -CD138-BV421 and -IgD-FITC (all from BD-Biosciences, San Jose, CA, USA)], and with mAb panel for Treg [anti-CD3-AlexaFlour700, -CD4-APC, -CD25-PE, -CD45-BV510, -CD127-BV421 (all from BD-Biosciences, San Jose, CA, USA)]. Following incubation, erythrocytes were lysed using FACS Lysing Solution (BD Biosciences, San Jose, CA, USA), and at least 10.000 cells were collected in CD45^+^ lymphocyte gate. The data were acquired on a NovoCyte flow cytometer (Agilent Technologies, USA) and analyzed using the NovoExpress operating system software (Agilent Technologies, USA).

### Peripheral blood mononuclear cell isolation

Peripheral blood mononuclear cells (PBMCs) were isolated from heparinized blood samples by density gradient centrifugation using Ficoll-Paque (Histopaque-1077; Biochrom, Cambridge, UK) at 2100 x rpm for 20 minutes, at room temperature. PBMCs were washed twice with phosphate-buffered saline (PBS) and suspended in a complete cell culture medium RPMI-1640 (c-RPMI) containing 10% Fetal Bovine Serum (Sigma Chem. Co., Germany), 1% L-glutamine (Sigma Chem. Co., Germany), 1% anti-mycotic and antibiotic solutions (Sigma Chem. Co., Germany).

### Flow cytometry analyses

#### ILCs

To analyze ILCs subsets, 3x10^6^ PBMCs/100 µL were labeled with anti-Lineage mix-FITC (anti-CD1a, -CD3, -CD4, -CD11c, -CD14, -CD16, CD19, -CD34, -CD94, -CD123, -CD303, -FcϵR1, -TCR alpha/beta, -TCR gamma/delta), -NKp44-PE, -CD161-PerCp/Cy5.5, -CD127-PE/Cy7, -CRTH2 (CD294)-APC, -c-kit (CD117)-BV421, -CD45-BV510 and Viability Dye-APC/CY7. All mAbs were purchased from Biolegend (San Jose, CA, USA). At least 1x10^6^ PBMCs were collected in lymphocyte gate. The samples were acquired on FACSAria II flow cytometer and analyzed using FACSDiva operating system software (BD Biosciences, San Jose, CA, USA).

### NK cell subsets

In order to analyze NK cell subsets and their receptor expression profile, PBMCs were labeled with anti-CD3-BV785, -CD16-BV570, -CD38-BV510, -CD56-BV711, -CD57-FITC, -NKG2A-PE, -NKG2D-PE/Cy7, -NKp30-BV421, -NKp44-PE/Cy7 and -NKp46-AlexaFluor700 mAbs (all from Biolegend, San Jose, CA, USA) for 20 minutes at room temperature in the dark. The data were collected and analyzed with NovoCyte flow cytometer with NovoExpress operating system software (Agilent Technologies, USA).

### Intracellular cytokine measurement

To measure the levels of intracellular cytokines of CD4^+^ and CD8^+^ T cells as well as NK cells, PBMCs (1×10^6^ cells/mL) were cultured for 4 hours with and without 2 μl Cell Stimulation Cocktail [containing PMA (40.5 μM), ionomycin (669.3 μM), and Brefeldin A (2.5 mg/ml)] (Biolegend, San Diego, USA). Following cell culture, PBMCs were washed with PBS and supernatants were discarded. Prior to the detection of intracellular cytokine levels, cell surface staining was performed using anti-CD3-BV785, -CD4-BV510, -CD8-PE/Cy5, -CD28-PE, -CD56-FITC and -CD57-BV711 mAbs (all from Biolegend, San Diego, USA). For intracellular staining, Fix&Cell Permeabilization Kit (Invitrogen, California, USA) was used according to the manufacturer’s protocol. Briefly, cells were fixed and then permeabilized together with addition of anti-IFN-γ-PE/Cy7, -TNF-α-APC/Cy7 and -IL-10-BV421 mAbs. Stained PBMCs were washed after the incubation and samples were analyzed on a NovoCyte flow cytometer running NovoExpress software (Agilent Technologies, USA).

### Measurement of NK cell cytotoxicity

Lysosomal–associated membrane protein-1 (LAMP-1 or CD107a), a glycosylated membrane protein primarily found in lysosomes, is a marker for degranulation of NK cells (26). Accordingly, CD107a is utilized as a cytotoxicity marker for functional assays. Therefore, anti-CD107a-PE-Cy5 mAb (Biolegend, San Jose, CA, USA) was added to the co-culture of PBMCs and human erythromyeloid leukemia cell line K562, with an effector/target (E:T) ratio of 10:1 for 4 hours at 37°C in the presence of anti-CD107a-PE/Cy5 mAb (Biolegend, San Jose, CA, USA). PBMCs alone were used as unstimulated control. Following the incubation, cells were stained with anti–CD3-Alexa Fluor700, -CD16-APC/Cy7 and CD56-BV650 mAbs (all from Biolegend, San Jose, CA, USA) for 20 minutes in the dark. To evaluate perforin and granzyme B content of PBMCs, samples were fixed and permeabilized according to the manufacturer protocol (Cytofix & Cytoperm Kit, Biolegend, San Jose, CA, USA), and then stained with intracellular anti-perforin-PerCP/Cy5.5 and -granzyme B-PE (Biolegend, San Jose, CA, USA) mAbs for 20 minutes. The samples were washed with PBS and were analyzed with NovoCyte flow cytometer running NovoExpress software (Agilent Technologies, USA).

### CFSE staining and cell culture

PBMCs (up to 2x10^7^ cells) suspended in RPMI-1640 medium were labeled with 1 μL of 5 mM CFSE solution (Thermo Fisher Scientific, USA) and incubated in dark for 6 minutes at 4°C. Cells were washed twice with PBS and were counted under light microscopy with trypan exclusion method. CFSE-labeled cells were cultured in 48-well flat bottom culture plates (NEST Biotechnology, China) for 120 hours at 37°C in 5% CO_2_ environment with the absence or existence of 5 µL/mL phytohemagglutinin (PHA, Thermo Fisher, USA). After the incubation, cell-culture supernatants were collected for future analysis and PBMCs were washed with PBS. After washing, PBMCs were re-suspended and 100 μL cell suspension was taken from the respective well (US: unstimulated, PHA: Phytohemagglutinin-stimulated) in 2 separate tubes for flow cytometry. Cells were labelled with anti-CD4-PE/Cy7 (BD Biosciences, USA) and -CD19-APC (BD Biosciences, USA) mAbs and incubated at dark for 20 minutes. After the incubation, cells were washed and re-suspended with FACS buffer. Samples were analyzed by a FACSCalibur flow cytometer running CellQuest software (BD Biosciences, San Jose, USA). The obtained data was evaluated as % proliferation. The proliferation percentages of total lymphocytes, CD4^+^ T cells and CD19^+^ B cells were evaluated.

### Cytokine measurement by multiplex assay

The levels of IL-1β, IL-18, IFN-α2, IFN-β, IFN-γ, IL-6, IL-10, TNF-α and Granulocyte/Macrophage Colony-Stimulating Factor (GM-CSF) were determined in serum samples using the Human Inflammation Panel 1 and the Anti-Virus Response Panel (LEGENDplex™, BioLegend, CA, USA). The cytokine levels of sera were measured by multiplex assay kit which is a bead-based immunoassay using flow cytometer according to manufacturer’s instructions. Data were measured with FACSCalibur flow cytometer (BD Biosciences, San Jose, USA) and analyzed using LEGENDplex™ v8.0 Data Analysis Software (Biolegend, San Jose, CA, USA).

The minimum detectable concentration (MDC) is the theoretical limit of detection calculated by applying a 5-parameter curve fitting algorithm. The values of assay sensitivity in serum samples are shown in **Supplement 1**. Assay sensitivity presented here is MDC + (2x Standard Deviation).

### SARS-CoV-2-specific antibody detection in sera

The presence of IgG-spike (IgG-SP), IgM-nucleocapsid (IgM-NP) and IgA spike (IgA-SP) antibodies specific for anti-SARS-CoV-2 in serum samples were evaluated by enzyme-linked immunosorbent assay (ELISA) method using the EUROIMMUN anti-SARS-CoV-2 kit (Lübeck, Germany) according to manufacturer’s protocol.

Photometric measurement (optical density/OD) of color intensity was done with a spectrophotometer device (ELx800, Bio-Tek, Instruments, INC. Belgium) at 450 nm wavelength (reference wavelength, 620-650 nm). The ratio/index value for the results was calculated (serum OD/calibrator OD). In obtained serum results, the ratio was accepted as negative if <0.8, limit if ≥0.8 - <1.1, and positive if ≥1.1.

### The measurement of complement component levels

The levels of C3a (Elabscience, Cat No. E-EL-H0818), C5a (Elabscience, Cat No. E-EL-H0190) were measured by ELISA according to manufacturer’s instructions. Optical densities were measured at 450 nm and concentrations were calculated by reference to the standard curves. The values of assay sensitivity in serum samples are shown in **Supplement 1**.

### HLA genotyping

All patients and controls were typed at the HLA Laboratory, Istanbul Faculty of Medicine which has accreditation to perform clinical HLA typing by the European Federation of Immunogenetics (EFI). All samples were typed at the HLA-A/B and -DRB1/loci by the sequence-specific oligonucleotide (PCR-SSO) method at low resolution. This typing method is the molecular equivalent of serological HLA-DR typing and identifies all two-digit specificities accurately including the presence of the super typical loci, not the DRB3 and DRB4 genes encoding DR52 and DR53, respectively. PCR was performed on a 9700-thermal cycle (Applied Biosystems, CA).

### Correlation network analysis

Correlation network analyses were performed to identify multidimensional differences between the mild, moderate, and severe categories of the patients. In this context, we only used the statistically significant measurements from pairwise comparisons followed by Bonferroni adjustment. The measurements were grouped according to their immunological origin (intracellular cytokines, T cell subsets, B cell subsets, ILCs, NK cell subsets, ELISA measurements, Multiplex, and lymphocyte proliferation) and Pearson correlation metrics were determined for each category after Z-score standardization of the data. Visualization of the data was carried out with a network plot with a correlation cutoff ± 0.7 to identify strong non-linear relationships between the measurements, groups, and prognostic categories.

### Statistical analysis

The data of the study were analyzed with the SPSS IBM 25.0 data package program and worked at 95% confidence limits. The normality test of the distribution of variables and measurements to be tested was calculated using Shapiro-Wilk test. The statistical analysis of independent and paired groups showing normal distribution were performed with Student-t test. Mann-Whitney U test was applied for the measurement variables when the numbers within the group were low and not normally distributed. Group tests of measurements before and after plasma treatment were analyzed with Wilcoxon test. Analysis of multiple groups was performed by ANOVA followed by Tukey’s test as *post-hoc*. Pearson’s correlation test was used for the correlation analysis. A value of p<0.05 was accepted as statistically significance limit. Graphics of the data were created by GraphPad Prism 9.0 program.

## Results

### Clinical features of patients

Median age of patients was 57 years (range; 21-71 years). As per the disease severity, 32% of patients had mild (n=11), 38% of patients had moderate (n=13), and 30% of patients had severe (n=10) disease. The clinical features of patients are summarized in **Table 1**. White blood cells (WBC), lymphocyte count, CRP, D-dimer, ferritin, and other biochemical parameters of the patients are given in **Table 2**. Correlation analyses were performed between these parameters and all data obtained from our findings. Significant correlations were stated in the text. There was a significant difference between the onset of the symptoms and the diagnosis time in severe patients (10 days) compared to mild (3 days) and moderate (5 days) patients (p=0.0003 and p=0.0003, respectively). Five of severe patients were receiving tocilizumab treatment before 1 or 3 days of collecting blood samples, and no differences were found in all parameter’s comparison between severe patients with or without tocilizumab treatment. Except patient with tocilizumab treatment, none of the patients have received dexamethasone or methylprednisolone.

**Table 1:** Demographic characteristics, initial signs and symptomatology.

n	Mild	Moderate	Severe
	11	13	10
**Gender**	8 males 3 females	9 males 4 females	8 males 2 females
**Age [Median (min-max)]**	56 (21 – 71)	58 (43 – 79)	65 (31 – 85)
**IP-10 Positivity**	n=1 (9%)	n=3 (23%)	n=1 (10%)
**Initial Symptoms**			
**Fatigue and myalgia**	n=5 (45%)	n=4 (30%)	n=4 (40%)
**Cough**	n=6 (54%)	n=8 (61%)	n=7 (70%)
**Fever**	n=5 (45%)	n=9 (69%)	n=7 (70%)
**Dyspnea**	n=7 (63%)	n=4 (30%)	n=5 (50%)
**Nausea**	None	n=1 (7%)	None
**Diarrhea**	n=2 (18%)	n=2 (15%)	n=1 (10%)
**Anosmia/Dysgeusia**	n=2 (18%)	n=3 (23%)	None
**Sputum**	None	None	n=1 (10%)
**Comorbid Conditions**			
**Hypertension**	n=1 (9%)	n=1 (7%)	n=2 (20%)
**Diabetes mellitus**	n=1 (9%)	n=2 (15%)	n=4 (40%)
**COPD or Asthma**	n=1 (9%)	n=2 (15%)	n=1 (10%)
**Coronary artery disease**	n=1 (9%)	n=2 (15%)	None
**Congestive heart failure**	None	None	None
**History of solid malignancy**	n=1 (9%)	n=2 (15%)	None
**History of Hematologic malignancy**	None	None	None
**Treatment**	Hydroxychloroquine, azithromycin	Hydroxychloroquine, azithromycin, favipravir	Hydroxychloroquine, azithromycin, favipravir, tociluzumab* dexamethasone*

*5 out of 10 severe patients were receiving tocilizumab and dexamethasone treatment.

**Table 2:** Biochemical parameters of patients.

	Mild median(min-max)		Moderate median(min-max)	Severe median(min-max)	p value	Reverences values
**White Blood Cells (10³/µL)**		6.98 (4.11 – 12.12)	8.01 (2.90 – 14.30)	6.14 (3.20 – 12.86)	p>0.05	4 – 10
**Lymphocytes (10³/µL)**		1.34 (0.29 – 3.50)	1.40 (0.79 – 2.21)	0.50 (0.40 – 0.69)	p<0.001	1.2 – 3.6
**Neutrophils (10³/µL)**		4.80 (2.27 – 11.80)	6.27 (1.50 – 12.50)	5.24 (2.00 – 11.52)	p>0.05	1.3 – 7
**Platelets (10³/µL)**		238 (158 – 368)	249 (153 – 529)	293 (29 – 640)	p>0.05	160 – 390
**CRP** **(mg/dL)**		6.62 (2.49 – 151)	46.32 (8.10 – 168.30)	60.52 (3.32 – 125.87)	p>0.05	0 – 5
**D-Dimer (µg/L)**		620 (270 – 15720)	820 (560 – 2570)	1900 (560 – 12010)	p=0.01	0.0 – 550
**Ferritin (ng/mL)**		376.5 (35.74 – 2300)	791 (215 – 3434)	755.5 (136.9 – 2492)	p=0.01	30 – 400 (for male) 13 – 150 (for female)
**Procalcitonin (ng/mL)**		0.07 (<0.2 – 44.18)	0.09 (0.03 – 1.06)	0.20 (0.20 – 6.11)	p=0.05	0 – 0.5

### Decreased ILC1 and increased ILC2 frequencies in mild and moderate patients

**Figure 1:**
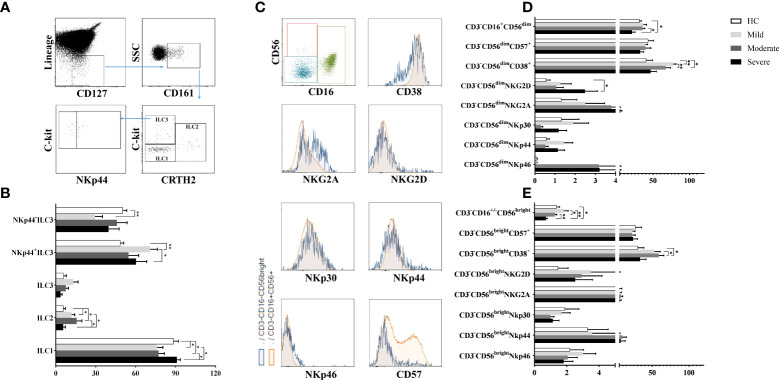
The evaluation of ILC and NK cell ratios of patients with COVID-19. **(A)** The gating strategy of ILC subsets were shown in the representative figure. Lineage^-^CD127^+^ cells were gated in CD45^+^ viable lymphocytes, and CD161^+^ cells were chosen as total ILCs. Total ILCs were divided into ILC2 and ILC3 subsets based on CRTH2 and c-Kit expression. **(B)** The frequencies of ILC subsets in patient groups and healthy controls were shown. **(C)** The gating strategy of NK cell subsets (CD3^-^CD16^+^CD56^dim^ and CD3^−^CD16^-/+^CD56^bright^ NK cells were gated in CD3^−^ cell population) and the expression of NK cell receptors/surface molecules were shown in the representative figure. **(D)** The expression levels of CD38, CD57 and NK cell receptors in CD3^−^CD16^+^CD56^dim^ and **(E)** CD3^−^CD16^-/+^CD56^bright^ NK cells were demonstrated in patient groups and healthy controls. *p<0.05, **p<0.001, HC, Healthy Controls.

Lymphocytes were gated on SSC/FSC dot-plot, and CD45^+^Lineage^−^CD127^+^CD161^+^ total ILCs were investigated for 3 subsets according to CRTH2 and c-kit expression: CD45^+^Lineage^−^CD127^+^CD161^+^CRTH2^−^c-kit^−^ cells for ILC1, CD45^+^Lineage^−^CD127^+^CD161^+^CRTH2^+^c-kit^+^ cells for ILC2 and CD45^+^Lineage^−^CD127^+^CD161^+^CRTH2^−^c-kit^+^ cells for ILC3 subset. ILC3 subpopulation was further subdivided based on NKp44 expression (27) (**Figure 1A**).

ILC1 frequencies were observed to be decreased in mild and moderate patients compared to healthy controls (p=0.04 and p=0.05; respectively) and also severe patients (p=0.01 and p=0.02; respectively). On the other hand, mild and moderate patients had increased ILC2 levels compared to healthy controls (p=0.05 and p=0.03; respectively) and also severe patients (p=0.05 and p=0.04; respectively) (**Figure 1B**).

There were no differences for ILC3 frequencies between patient groups and healthy controls. However, increased NKp44^+^ ILC3 subset were found in mild patients compared to healthy controls (p=0.003) and severe patients (p=0.02) whereas NKp44^−^ ILC3 cells were decreased in mild patients compared to healthy controls (p=0.004) (**Figure 1B**).

Although ILC1 levels were decreased in mild COVID-19 patients, ILC2 and IL-23 expressing NKp44^+^ ILC3 expression increased in moderate patients. Similar to mild patients, ILC1 cell subset was decreased, however ILC2 subset increased in moderate patients. There was no differences ILC subset between healthy controls and severe patients.

### Reduced NK cell subsets in severe patients

To investigate the involvement of NK cells in the pathogenesis of COVID-19, CD3^−^CD16^+^CD56^dim^ and CD3^−^CD16^−/+^CD56^bright^ NK cell subsets and their NKp30, NKp44, NKp46, NKG2A, NKG2D, CD57 and CD38 expression levels were detected by flow cytometry. The gating strategy of NK cell subsets is shown in **Figure 1C**. CD3^−^CD16^+^CD56^dim^ frequencies of severe patients, which were the lowest among all donor groups, were found to be significantly decreased compared to moderate patients and healthy controls (p=0.021, p=0.048; respectively) (**Figure 1D**). The expression levels of CD3^−^CD16^−/+^CD56^bright^ NK cells of severe patients were also the lowest which were significantly decreased in comparison with mild and moderate patients as well as healthy controls (p=0.002, p=0.049, p=0.002; respectively) (**Figure 1E**). CD3^−^CD16^−/+^CD56^bright^ NK cell levels of moderate patients were decreased compared to mild patients (p=0.049).

NKG2D expression of CD3^−^CD16^+^CD56^dim^ NK cells were found to be increased in severe patients compared to healthy controls (p=0.05) (**Figure 1D**). However, no significant difference was found in NKG2A, NKp30, NKp44 and NKp46 expression levels of both NK cell subsets of patient groups and healthy controls. On the other hand, increased CD38 levels of CD3^−^CD16^+^CD56^dim^ NK cells were revealed in mild and moderate patients compared to healthy controls (p=0.004 and p=0.02; respectively). CD38^+^CD3^−^CD16^+^CD56^dim^ NK cells of moderate patients were also increased in comparison with mild patients (p=0.003) (**Figure 1D**). CD38 expression levels were observed to be increased in CD3^−^CD16^−/+^CD56^bright^ NK cells in contrast to CD3^−^CD16^+^CD56^dim^ NK cells dependent on disease severity. CD3^−^CD16^−/+^CD56^bright^ NK cells were significantly increased in moderate patients compared to healthy controls and severe patients (p=0.01 and p=0.02; respectively) (**Figure 1E**). CD3^−^CD16^+^CD56^dim^ and CD3^−^CD16^−/+^CD56^bright^ NK cell frequencies showed a decrease with disease severity while severe patients had the lowest levels among all groups.

### Decreased IFN-γ levels in contrast to increased IL-10 levels of NK cells in severe patients

**Figure 2:**
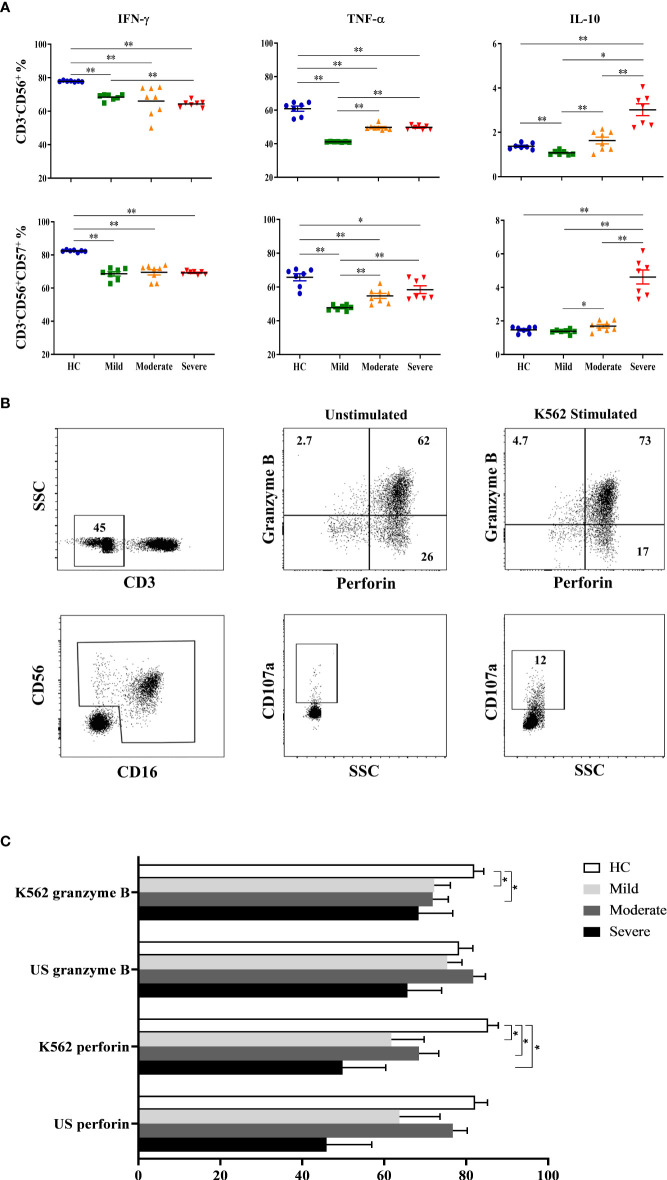
Intracellular cytokine levels and cytotoxic capacity of NK cells in COVID-19 patients. **(A)** IFN-γ, TNF-α and IL-10 levels of CD3^−^CD56^+^ and CD3^−^CD56^+^CD57^+^ NK cells. **(B)** To analyze the capacity of NK cell cytotoxicity, PBMCs were cultured with the absence and existence of K562 cells. Granzyme B, perforin and CD107a levels of NK cells were measured to evaluate the cytotoxicity. CD3^−^CD16^+^CD56^+^ cells were gated as total NK cells. Granzyme B, perforin and CD107a expression of total NK cells were shown in the representative figure. **(C)** Perforin and granzyme B expression levels of patients and healthy controls, at the presence and absence of K562 cells. *p<0.05. **p<0.001, HC, Healthy Controls.

Following the detection of NK cell subsets and their receptor expression, NK cells were investigated for their cytokine content. In addition to CD3^-^CD56^+^ cells which were considered as total NK cells, CD57-expressing CD3^−^CD56^+^ NK cells were also analyzed for IFN-γ, TNF-α and IL-10 secretion. Intracellular cytokine levels were measured following cell culture with the absence and existence of stimuli. IFN-γ levels were found to be decreased in all patient groups, especially in severe patients (**Figure 2A**). IFN-γ content of CD3^−^CD56^+^ NK cells of mild, moderate and severe patients were significantly lower than healthy controls (p=0.002, p=0.001, p=0.002; respectively). Severe patients also had lower IFN-γ levels compared to mild patients (p=0.006). CD57-expressing CD3^−^CD56^+^ NK cells of all patient groups displayed a similar profile with those of CD3^−^CD56^+^ NK cells. IFN-γ levels of CD3^−^CD56^+^CD57^+^ NK cells were significantly decreased in mild, moderate and severe patients compared with healthy controls (p=0.002, p=0.001, p=0.002; respectively) (**Figure 2A**).

Similar to IFN-γ levels, diminished TNF-α levels of CD3^−^CD56^+^ NK cells were also detected in all patient groups. TNF-α content of CD3^−^CD56^+^ NK cells were significantly decreased in mild, moderate, and severe patients in comparison with healthy controls (p=0.002, p=0.001, p=0.002; respectively) (**Figure 2A**). However, the lowest TNF-α level was observed in mild patients which were significantly lower than moderate and severe patients (p=0.001, p=0.002; respectively). Parallel to CD3^−^CD56^+^ NK cells, CD3^−^CD56^+^CD57^+^ NK cells of patient groups also had reduced TNF-α levels which were significantly lower in mild, moderate, and severe patients than healthy controls (p=0.002, p=0.008, p=0.018; respectively) (**Figure 2A**). Moreover, TNF-α content of CD3^−^CD56^+^CD57^+^ NK cells were significantly decreased in mild patients compared to moderate and severe patients (p=0.003, p=0.002; respectively). The observation that mild patients had the lowest TNF-α levels among the other patient groups might indicate that TNF-α plays a more important role in the early phase rather than in severe patients.

In contrast to decreased levels of pro-inflammatory IFN-γ and TNF-α in patient groups, anti-inflammatory cytokine IL-10 was observed to be increased especially in the severe phase of the disease. IL-10 levels of CD3^−^CD56^+^ NK cells were significantly higher in severe patients than mild and moderate patients as well as healthy controls (p=0.002, p=0.001, p=0.002; respectively) (**Figure 2A**). Like TNF-α, IL-10 content of CD3^−^CD56^+^ NK cells in mild patients were also detected to be lowest among donor groups and significantly decreased in comparison with moderate and severe patients as well as healthy controls (p=0.012, p=0.002, p=0.003; respectively). CD3^−^CD56^+^CD57^+^ NK cells of severe patients had also increased levels of IL-10 compared with mild and moderate patients and healthy controls (p=0.002, p=0.001, p=0.002; respectively) (**Figure 2A**). In addition, IL-10 levels of CD3^−^CD56^+^CD57^+^ NK cells in mild patients were significantly lower than moderate patients (p=0.028). Overall, IL-10 levels observed in from mild to severe patients were remarkable, which might indicate that IL-10 is elevated in severe patients.

### Decreased cytotoxic capacity of NK cells in patients

To analyze the capacity of NK cell cytotoxicity, PBMCs were incubated with K562 human erythromyeloid leukemia cells **Figure 2B**. CD107a expression levels of total NK cells were observed to be decreased in all patient groups compared to healthy controls, though significance was only found in moderate patients (p=0.04) (**Figure 2C**). No correlation was observed for CD107a expression when comparing IP-10, lymphopenia status, D-dimer, CRP or ferritin levels of patient groups. Perforin expression levels of total NK cells were significantly diminished in mild, moderate and severe patients in comparison with healthy controls (p=0.02, p=0.008 and p=0.01; respectively). Similarly, granzyme B expression levels of NK cells in mild and moderate patients were reduced compared to healthy controls (p=0.05 and p=0.04; respectively) (**Figure 2C**).

### Increased plasmablast and plasma cells in COVID-19 patients

**Figure 3:**
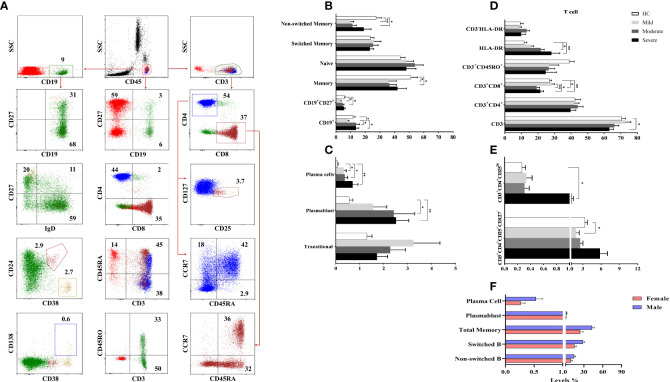
The frequencies of B and T cell subsets. **(A)** The gating strategy of T and B cell subsets were shown in the representative figure. **(B, C)** The frequencies of B and **(D, E)** T cell subsets were shown in patient groups and healthy controls. **(F)** The differences of B cell subsets between male and female patients with COVID-19. *p<0.05, **p<0.001, HC, Healthy Controls.

To determine the involvement of CD19^+^ B cells in the pathogenesis of COVID-19, circulating CD19^+^ (total B cell), CD19^+^IgD^+^CD27^−^ (naive B cell), CD19^+^CD27^+^ (total memory B cell), CD19^+^IgD^+^CD27^+^ (non-switched B cell), CD19^+^IgD^−^CD27^+^ (switched B cell), CD19^+^CD38^++^CD138^+^ (plasma cell) and CD19^+^CD38^+^CD24^−^ (plasmablast) were analyzed by flow cytometry (**Figure 3A**).

There were no significant differences for the CD19^+^ B cells in moderate and severe patients compared to healthy controls. However, mild patients had decreased CD19^+^ B cells compared to healthy controls, moderate and severe patient groups (p=0.02, p=0.03, and p=0.01; respectively). The frequency of naive B cells was not different between patient groups and healthy controls, but diminished memory B cells were found in mild and moderate patients compared to healthy controls (p=0.04 and p=0.04; respectively) (**Figure 3B**). There was a positive correlation only between memory B cells and D-dimer levels (p<0.05, R: 0.349).

Non-switched B cell levels were reduced in mild and moderate patient groups compared to healthy controls (p=0.001 and p=0.001; respectively). The frequency of switched B cells was similar in patient groups and healthy controls. Elevated plasma cell frequency was found in mild, moderate and severe patient groups compared to healthy controls (p=0.04, p=0.04 and p=0.004; respectively). Similarly, plasmablast were increased in all patient groups except mild patients compared to healthy controls (p=0.05 and p=0.005; respectively) (**Figure 3C**).

Non-switched, switched, total memory B cells, plasmablast and plasma cells were increased in male patients compared to female patients (p=0.05, p=0.001, p=0.001, p=0.03 and p=0.02; respectively) (**Figure 3F**).

### Contribution of T cell subsets in COVID-19 immunopathology

To determine the involvement of T cells in the pathogenesis of COVID-19, circulating CD3^+^ total T cells, CD3^+^CD4^+^ and CD3^+^CD8^+^ T cells, CD3^+^CD45RA^+^, CD3^+^CD45RO^+^, CD3^+^HLA-DR, CD3^+^CD4^+^CD25^+^CD127^−^, CD3^+^CD4^+^CD25^++^ cells as well as CD45RA^+^CCR7^+^ (naive), CD45RA^+^CCR7^−^ (exhausted), CD45RA^−^CCR7^−^ (effector memory), CD45RA^−^CCR7^+^ (central memory) cells in CD3^+^CD4^+^ and CD3^+^CD8^+^ T cells were analyzed by flow cytometry (**Figure 3A**).

There were no differences in CD3^+^ T cells in mild and moderate patients compared to healthy controls whereas CD3^+^ T cells were decreased in severe patients compared to healthy controls (p=0.03). A negative correlation was found between CD3^+^ T cells and D-dimer levels (p<0.05, R: -0.391).

The levels of CD3^+^CD4^+^, CD3^+^CD45RA^+^ and CD3^+^CD45RO^+^ cells were similar in all patients and healthy controls. On the other hand, CD3^+^CD8^+^ T cells of moderate and severe patients were decreased compared to mild patients (p=0.002 and p=0.02; respectively) as well as healthy controls (p=0.001 and p=0.01; respectively). Expression of HLA-DR in CD3^+^ T cells was significantly increased in moderate and severe patient groups compared to healthy controls (p=0.009 and p=0.03; respectively) (**Figure 3D**).

No differences were found between naive, exhausted, effector and central memory cells of CD4^+^ or CD8^+^ T cell subsets between patient groups and healthy controls. CD3^+^CD4^+^CD25^++^ cells were significantly increased in severe patients compared to healthy controls (p=0.01), however CD3^+^CD4^+^CD25^+^CD127^−^ T regulatory cells were decreased in mild patients compared to healthy controls (p=0.05) (**Figure 3E**).

### Increased IL-10 levels of T cell subsets in moderate and severe patients

Intracellular cytokine measurement was performed following the detection of surface molecules on T cell subsets. PBMCs cultured at unstimulated (data not shown) and stimulated conditions were stained for evaluation of IFN-γ, TNF-α and IL-10 contents of T cell subsets.

**Figure 4:**
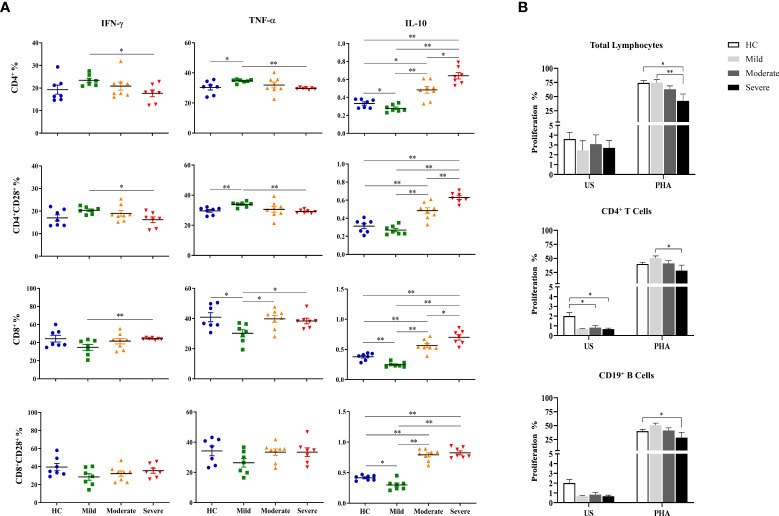
Determination of intracellular cytokine levels in CD4^+^ and CD8^+^ T cells and the proliferative capacities of lymphocytes. **(A)** IFN-γ, TNF-α and IL-10 levels of CD4^+^, CD4^+^CD28^+^, CD8^+^ and CD8^+^CD28^+^ T cells. **(B)** The proliferative capacity of total lymphocytes. T and B cells were shown in patient groups and healthy controls. *p<0.05, **p<0.001, HC, Healthy Controls.

Although IFN-γ levels of CD4^+^ T cells of COVID-19 patients did not have a significant difference with that of healthy controls, they were observed to be decreased from mild to severe patients. IFN-γ content of CD4^+^ T cells in mild patients was significantly higher than severe patients (p=0.013) (**Figure 4A**). A similar profile was shown in CD4^+^CD28^+^ T cells which had significantly increased IFN-γ levels in mild patients in comparison with severe patients (p=0.018). In contrast to CD4^+^ and CD4^+^CD28^+^ T cells, IFN-γ content of CD8^+^ as well as CD8^+^CD28^+^ T cells were observed to be increased from mild to severe patients, though there was no significant difference among CD8^+^CD28^+^ T cells of patients and healthy controls. IFN-γ levels of CD8^+^ T cells were significantly increased in severe patients compared to mild patients (p=0.003) (**Figure 4A**).

T cells exhibited a very similar profile to that of IFN-γ with decreased TNF-α levels in CD4^+^ and CD4^+^CD28^+^ T cells but increased in CD8^+^ and CD8^+^CD28^+^ T cells with disease progression. TNF-α levels of CD4^+^ and CD4^+^CD28^+^ T cells of mild patients were significantly increased compared to severe patients (p=0.002, p=0.002; respectively) as well as healthy controls (p=0.035, p=0.003; respectively) (**Figure 4A**). In contrast, TNF-α^+^CD8^+^ T cells of mild patients demonstrated to be significantly lower than moderate and severe patients besides healthy controls (p=0.021, p=0.013, p=0.048; respectively). Although TNF-α levels in CD8^+^CD28^+^ T cells of mild patients also tended to be decreased, no significant difference was observed in comparison with the other donor groups (**Figure 4A**).

In contrast to IFN-γ and TNF-α, IL-10 levels of both CD4^+^ and CD8^+^ T cells and of their CD28-expressing subgroups were increased by disease progression. IL-10 content of CD4^+^ and CD4^+^CD28^+^ T cells in severe patients were significantly higher than mild (p=0.002, p=0.002, respectively) and moderate patients (p=0.011, p=0.005; respectively) as well as healthy controls (p=0.002, p=0.002; respectively) (**Figure 4A**). Moderate patients were observed to have increased IL-10 levels of CD4^+^ and CD4^+^CD28^+^ T cells compared to mild patients (p=0.002, p=0.002, respectively) and healthy controls (p=0.017, p=0.006; respectively) (**Figure 4A**). Among all donor groups, mild patients showed the lowest IL-10 levels in CD4^+^ and CD8^+^ T cells and their CD28-expressing subgroups, however, there was no significant difference in IL-10^+^CD4^+^CD28^+^ T cells compared to healthy controls. IL-10 levels of CD4^+^ T cells in mild patients were significantly lower than healthy controls (p=0.04) (**Figure 4A**).

Notably, elevation of IL-10 levels with disease severity was not only observed in CD4^+^ and CD4^+^CD28^+^ T cells, but also in CD8^+^ and CD8^+^CD28^+^ T cells, especially of severe patients. IL-10 content of CD8^+^ and CD8^+^CD28^+^ T cells was detected to be significantly increased in severe patients compared to mild patients (p=0.002, p=0.002; respectively) and healthy controls (p=0.002, p=0.002; respectively) (**Figure 4A**). There was also a significant increase in IL-10 levels of CD8^+^ T cells in severe patients in comparison with moderate patients (p=0.042) whereas their IL-10^+^CD8^+^CD28^+^ T cell frequencies were similar. Like CD4^+^ and CD4^+^CD28^+^ T cells, CD8^+^ and CD8^+^CD28^+^ T cells were demonstrated to have significantly greater levels of IL-10 in moderate patients than mild patients (p=0.001, p=0.001; respectively) and healthy controls (p=0.005, p=0.001; respectively). Having the lowest levels, mild patients had significantly reduced IL-10 content in CD8^+^ and CD8^+^CD28^+^ T cells compared to healthy controls (p=0.004, p=0.015; respectively) (**Figure 4A**) as well as moderate and severe patients mentioned above. It is notable that IL-10 levels of both CD4^+^ and CD8^+^ T cells and their CD28-expressing subgroups were in accordance with those of CD3^−^CD56^+^ and CD3^−^CD56^+^CD57^+^ NK cells.

### Decreased proliferative responses in severe COVID-19 patients

The proliferative responses of total lymphocytes, CD4^+^ T and CD19^+^ B cells were evaluated by flow cytometric CFSE dilution method following culture of PBMCs with the absence and presence of PHA, for 120 hours.

Spontaneous proliferation levels of total lymphocytes were similar among mild, moderate and severe patients as well as healthy controls. PHA-induced proliferation was observed to be decreased with the advancement of disease severity. Severe patients had significantly diminished proliferative response to PHA when compared with mild patients and healthy controls (p=0.01 and p=0.02; respectively) (**Figure 4B**). No significant differences of proliferation neither with the absence nor the existence of PHA were observed between genders. PHA-induced lymphocyte proliferation was observed to be significantly diminished in patients with IP-10 positivity (p=0.005). Patients with lymphopenia had significantly lower proliferative responses to PHA stimulation (p=0.008).

When proliferative responses of CD4^+^ T cells were investigated, the spontaneous proliferation of moderate and severe patients were significantly decreased in comparison with that of healthy controls (p=0.15 and p=0.04; respectively) (**Figure 4B**). PHA-induced CD4^+^ T cell proliferation levels were observed to be decreased in concordance with increased disease severity and severe patients had significantly diminished proliferative responses when compared with mild patients (p=0.05). When IP-10 positivity was considered, PHA responses of CD4^+^ T cells were significantly diminished in IP-10 positive patients when compared with that of IP-10 negative patients (p<0.001). However, patients with lymphopenia also had diminished CD4^+^ T cell proliferative responses under PHA stimulation (p=0.04). Similarly, proliferative responses of CD4^+^ T cells were decreased in moderate patients with lymphopenia (p=0.05).

On the other hand, there was a negative correlation between the proliferative responses of CD4^+^ T cells and D-dimer levels (p=0.02, R= -0.384) (**Figure 4B**).

No significant difference was detected in spontaneous proliferation of CD19^+^ B cells between patient groups and healthy controls. However, mitogen response of CD19^+^ B cells are heterogeneous, whereas proliferative response of severe patients was significantly reduced compared to healthy controls (p=0.05) (**Figure 4B**).

According to the findings showing decreased proliferative responses in patients with lymphopenia compared to those without lymphopenia, it has been demonstrated that lymphopenia status might affect the proliferative responses.

### Increased pro-inflammatory cytokines in severe COVID-19 patients

**Figure 5:**
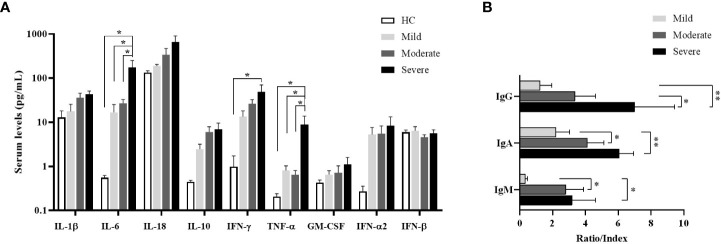
Cytokine measurement by multiplex assay in serum samples and determination of SARS-CoV-2-specific antibody ratios. **(A)** IL-1β, IL-6, IL-18, IL-10, IFN-γ, TNF-α, GM-CSF, IFN-α2 and IFN-β levels in serum of patient groups and healthy controls were determined by multiplex assay. Statistical analysis was performed by ANOVA followed by Tukey’s test. **(B)** Anti-SARS-CoV-2-specific IgM (nucleocapsid), IgG (spike) and IgA (spike) concentrations in serum samples of patient groups. *p<0.05, **p<0.001, HC, Healthy Controls.

The levels of IL-1β, IL-6, IL-18, IL-10, IFN-γ, TNF-α, GM-CSF, IFN-α2 and IFN-β were detected by multiplex assay in serum samples of all donor groups. IL-6 levels of all patient groups were tended to be higher than healthy controls, which were highest in severe patients, and were significantly increased in severe patients compared to mild and moderate patients as well as healthy controls (p=0.015, p=0.028, p=0.012; respectively) (**Figure 5A**). Similarly, TNF-α levels were significantly increased in severe patients in comparison with mild and moderate patients as well as healthy controls (p=0.046, p=0.040, p=0.047; respectively). IFN-γ levels in serum samples of all patient groups tended to be greater than healthy controls, which were significantly elevated in severe patients compared to healthy controls (p=0.033). In addition, IL-10 and IFN-α2 were detected to be higher in all patients than healthy controls without significance.

### Increased SARS-CoV-2-specific antibody levels in severe patients

To understand the antibody responses following SARS-CoV-2 infection, IgM-NP, IgG-SP, and IgA-SP were analyzed in serum samples of patient groups (**Figure 5B**). IgG-SP positivity was found in 14 patients (3 of mild, 6 of moderate, 5 of severe patients). Although IgG-SP levels were increased in proportion to the severity of the disease, elevated IgG-SP levels were only found in severe patients compared to mild patients (p=0.007). However, compared to moderate patients, the increase in IgG-SP levels of severe patients was also at the limit of statistical significance (p=0.07).

IgA-SP positivity was detected in 22 patients (5 of mild, 10 of moderate and 5 of severe patients). IgA-SP levels were increased in severe patients compared to mild and moderate patients (p=0.002 and p=0.04; respectively).

IgM was measured as positive only in 11 patients (2 of mild, 6 of moderate and 3 of severe patients). IgM levels were increased in severe and moderate patients compared to mild patients (p=0.05 and p=0.03; respectively).

### Serum complements breakdown product levels

It is known that complement proteins levels, especially C3a and C5a, have a role in inflammatory response of anti-viral immunity (28). Thus, C3a and C5a levels in serum samples were determined. There were no differences in C3a and C5a sera levels between patient groups and healthy controls (data not shown).

### HLA frequencies in COVID-19 patients

HLA-A*02 (n=24, 40%), HLA-A*01 (n=7, 11.7%), HLA-B*50 (n=6, 10%), HLA-B*35 and HLA-B*38 (n=5, 8.3%), HLA-DRB1*07 (n=12, 20%), and HLA- DRB1*04 (n=9, 15%) were most identified in all COVID-19 patients (n=30). HLA-A*24 (n=7, 35%), HLA-A*03 (n=3, 15%), HLA-B*35 and HLA-B*51 (n=4, 20%), HLA-B*15 (n=3, 15%), HLA- DRB1*13 (n=5, 25%), and HLA- DRB1*03, HLA- DRB1*04, HLA- DRB1*11 (n=3, 15%) were most commonly identified in healthy controls (n=10).

However, the most common HLA alleles are HLA-A*02 (n=9, 56.3%) and HLA-A*33 (n=3, 18.8%), HLA-B*14, HLA-B*27, HLA-B*44 and HLA-B*49 (n=2, 12.5%), HLA- DRB1*04 and HLA-DRB1*11 (n=3, 18.8%) in mild patients (n=8), HLA-A*02 (n=8, 33.3%), HLA-A*24 (n=3, 12.5%), HLA-B*15, HLA-B*18, HLA-B*38 and HLA-B*39 (n=3, 12.5%), HLA- DRB1*13 (n=5, 20.8%) and HLA-DRB1*03, HLA-DRB1*04, HLA-DRB1*07 (n=3, 12.5%) in moderate patients (n=12), and HLA-A*02 (n=7.35%), HLA-A*01 (n=4.20%), HLA-B*50 (n=5, 25%), HLA-B*35 (n=3, 15%), HLA-DRB1*07 (n=7, 35%), and HLA-DRB1*04, HLA-DRB1*15 (n=3, 15%) in severe patients (n=10).

### Correlation network analysis

**Figure 6:**
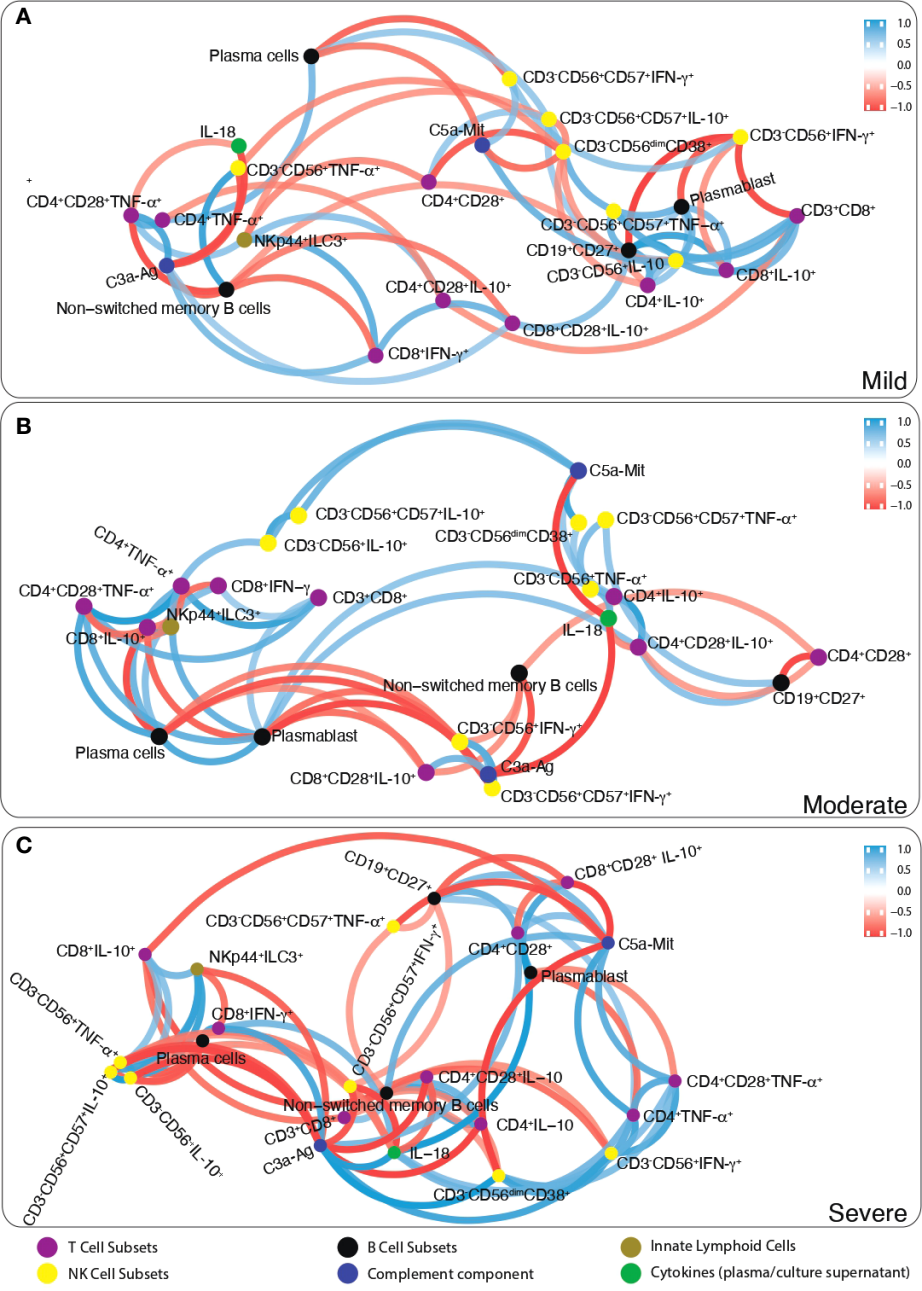
The network analysis of immune parameters in COVID-19 patients. Correlation network plot of ILCs, NK cells, T and B lymphocytes, complement components and cytokines levels of **(A)** mild, **(B)** moderate and **(C)** severe patients. Blue lines between parameters indicate positive correlations whereas red lines indicate negative correlations. The significance of correlations increases in proportion with increased boldness of line colors.

Firstly, the correlation number of each parameter with other parameters was determined. Then, the most correlated parameters with others (>6 correlation) were chosen. Correlation analysis data of immunological parameters in mild, moderate and severe patients were included as a supplement. According to this approach, while CD19^+^CD27^+^ memory B cells, plasmablasts and anti-inflammatory cytokine IL-10 in CD8^+^, CD4 and NK cells were positively correlated in mild patients; non-switched memory B cells were negatively correlated CD3^−^CD56^+^CD38^+^ NK, CD3^−^CD56^+^CD57^+^IFN-γ^+^ NK and IL-10 expressing CD8 and NK cells in severe patients (**Figure 6**). These parameters were checked with pairwise comparisons, CD19^+^CD27^+^ memory and non-switched memory B cells were increased from mild to severe patients and correlated with increased SARS-CoV-2 IgA and IgG levels from mild to severe patients. Additionally, IL-10-expressing CD4, CD8 and NK cell were increased from mild to severe, CD4^+^TNF-α^+^ and CD3^−^CD56^+^IFN-γ^+^ were decreased in severe compared to mild. These results might suggest that increased anti-inflammatory and decreased inflammatory response were correlated with disease severity.

## Discussion

The SARS-CoV-2 virus has spread around the world in a short time since the first case of COVID-19 was reported in Wuhan in December 2019 (29). It has become a pandemic that continues to deeply affect social life, especially the health system, in all countries. The studies showed that the presence of comorbidity like diabetes mellitus, hypertension are associated with poor clinical outcome in COVID-19 patients (30). In our study, the presence of comorbidties were seen increased from mild to severe patients, such as incidence of diabetes mellitus was 9% in mild but 40% in severe patients. Although the use of vaccines developed against COVID-19 allows to overcome the disease with mild symptoms, the success of the drugs used in the treatment in COVID-19 infection has not yet been clarified (31). In particular, it is very important to observe the role of immune system cells and the change in the functions of these cells with the effects of drug treatments used in COVID-19 infection. Since the first case was observed, published data on the immunopathogenesis of COVID-19 from various centers differ from each other. In this study, the role of immune cells in the pathogenesis of COVID-19 was aimed to be clarified by analyzing the patients in the period when the first peak of the pandemic was observed in our country.

NK cells, divided into CD3^−^CD16^+^CD56^dim^ and CD3^−^CD16^-/+^CD56^bright^ subgroups, have an important role in the lysis of the virus-infected cells via secreting perforin and granzyme, and in stimulating the activities of other immune cells through cytokine production (8). In our study, there was no difference in NK subgroups in mild patients who did not require hospitalization compared to healthy controls, while diminished NK cells was observed in moderate and severe patients. CD3^−^CD16^-/+^CD56^bright^ NK cells were shown to be significantly reduced in severe patients. Similarly, the perforin and granzyme B content of NK cells were significantly decreased from mild to severe patients. These data might indicate that patients with low NK cell frequency and cytotoxic capacity might be associated with increased disease severity. In the literature, NK cell counts and perforin, granzyme B levels were reported to be decreased in COVID-19 patients (32). On the other hand, some studies showed that high NK cells and cytotoxic enzymes were associated especially with prolonged hospitalization (33, 34). A meta-analysis data of COVID-19 studies showed that NK cell counts were significantly reduced in patients with severe disease (6). In addition, people hospitalized with COVID-19 were reported to have 1.78 times diminished ILCs and 2.31 times less NK cells than healthy controls (34).

According to the analysis of NK cell cytokine contents in our study, pro-inflammatory cytokines such as IFN-γ and TNF-α were decreased compared to healthy controls, while the anti-inflammatory cytokine IL-10 was increased in severe patients compared to both healthy controls and the other patient groups. This result might suggest that the capacity of NK cells to stimulate macrophages and other phagocytic cells is reduced in COVID-19 patients. CD57 is a very useful marker of NK cell maturation, identifying cells with potent cytotoxic potential but decreased sensitivity to cytokines and reduced replicative potential (35). CD57 have also a role in migration of NK cells to inflamed tissue (36). Our data showed that, IFN-γ, TNF-α and IL-10 cytokine content of CD57-expressing NK cells were similar. A study in COVID-19 pathogenesis about NK cells indicated that CD57-expressing NK cells were increased in COVID-19 patients (35). In our study, CD57-expressing NK cells were analyzed in mild, moderate and severe patients and no differences were observed among patient groups. This might be due to the difference in patient selection criteria or treatment status. Unlike this sudy, increased anti-inflammatory and decreased pro-inflammatory cytokines were detected in CD57-expressing NK cells of patients (35).

It is known that ILC2s also play a role in inflammation in the lungs and airways after exposure to viruses and allergens. In particular, ILC2 cells secrete various cytokines such as IL-4, IL-5 and IL-13 to stimulate mucus production, bronchial hyperactivity, airway remodeling and tissue repair (37). In our study, decreased ILC1 and increased ILC2 ratios in mild and moderate patients were observed. Considering that SARS-CoV-2 causes pneumonia by affecting the lungs, this finding suggest that increased ratios of ILC2s might have a protective role in people with mild and moderate disease.

Studies on COVID-19 showed that CD4^+^ and CD8^+^ T lymphocyte ratios decrease with the severity of the disease (6, 38). Similarly, in our study, CD3^+^ and CD3^+^CD8^+^ T lymphocyte rates were found to be decreased in moderate and severe patient groups requiring hospitalization. The decrease of CD8^+^ T lymphocytes together with NK cells which play a role in the response to viral infections might be an important parameter to predict hospitalization and the clinical outcome of the disease.

IL-6, TNF-α and IFN-γ levels were elevated in all patients in relation to disease severity in this study. Similarly, previous studies have indicated that these proinflammatory cytokines are increased in sera of severe patients. Thus these cytokines might be independent and significant predictor biomarkers of clinical outcome of COVID-19 (39, 40). Considered with our intracellular cytokine data, it is thought that the increased serum TNF-α and IFN-γ in severe patients may originate from macrophage and ILCls.

As in NK cells, pro-inflammatory TNF-α and IFN-γ levels were observed to be decreased whereas IL-10 levels were increased in CD4^+^ T lymphocytes in relation with severity of the disease. According to our data, elevated frequencies of Treg cells seen in severe patients also support the findings that immune responses are directed to an anti-inflammatory direction. It has been reported that the depletion of Tregs might increase the production of proinflammatory cytokines which cause lung damage by stimulation of proinflammatory immune cells and cytokine storm in severe patients (41). The elevated IL-10 levels in NK cells as well as CD4^+^ and CD8^+^ T cells of severe patients might be also considered as a protective response against the harmful effect of cytokine storm seen in COVID-19.

It was demonstrated that the levels of SARS-CoV-2-specific IgM, IgG and IgA antibodies were increased in association with higher disease severity. Congruently, plasmablasts and plasma cells, which are primarily responsible for antibody production, were increased in moderate and severe patients in our study population. Thus SARS-specific antibody levels might be another predictive biomarker of severity in COVID-19.

Tocilizumab was used in COVID-19 patients with a risk of cytokine storm (42), and the studies showed that tocilizumab was associated with a lower relative risk of mortality (43). The studies showed that tocilizumab has a low effect on parameters such as D-dimer and lymphocytes, but influences on CRP and ferritin enough to be clinically important (44). A meta-analysis study indicated that tocilizumab has an effect on the number of lymphocytes 5-8 days after administration (44). Similarly, a study focused on patients with SARS-CoV-2 infection, was determined that absolute lymphocyte counts did not change in the daily follow-up performed during the 14 day after tocilizumab treatment (45). In this study, 5 out of 10 severe patients were receiving tocilizumab treatment and no differences were found in all parameters’ comparison between severe patients with or without tocilizumab treatment. Thus, plausibly collection of blood samples within 1-3 days after tocilizumab administration (within the first 24 hours in 4 patients, within 72 hours in one patient) and the low number of samples might be the reason underlying the lack of difference between pre- and post-CP samples. Nevertheless, it should be noted that mild, moderate and severe patients were receiving different treatment types in our study and this may have had an impact on our results. Due to low number per treatment type and low statistical power, we could not delineate the impact of treatment types on immune responses. More importantly, there was a strong overlap between treatment types and severity of COVID-19 (e.g. tocilizumab was used for severe patients and kinin was used for mild patients mostly), which made it impossible to assess whether observed immunological differences were due to treatment or disease severity and a different study design will be required to address this question in future studies.

HLA genes, which are important targets for natural selection, were the most polymorphic genes in the human genome (46). HLA molecules serve as an important factor for regulating the outcome of various infections in the host organism and stand out as a leading candidate for disease susceptibility, and it has been recognized that HLA molecules play a central role in antigen presentation and additionally provide modulation of NK cell activity (8, 47). All these features of both HLA class I and class II highlight the importance of properly regulated HLA expression for the control of immune response in health and disease (48). HLA plays a pivotal role in the immune response to pathogens so HLA variation may be associated with SARS-CoV-2 infection (49). To show that alleles that may reflect a susceptibility to SARS-CoV-2 infection, HLA allel frequency distribution in COVID-19 patients were analyzed. While HLA-A*02 is the most investigated allele (49), it has been showed that there is an association between HLA-DRB*04 and symptomatic infections on COVID-19 (50). Consistent with these results, HLA-A*02 and HLA-DRB1*04 were most common alleles in all patient groups in our study. Moreover, allele frequency of HLA-A*01 and HLA-DRB1*07 were mostly seen in severe patients. On the other hand, HLA-A*03, HLA-B*51, and HLA-DRB1*15 were mostly seen in healthy controls, while not seen in patients. The increased frequencies may contribute to identify potential markers of susceptibility to the disease, although the distribution of HLA alleles and their association with COVID-19 is very limited in our study. The small sample size might represent a risk for a false positive finding. According to the evaluation of the literature, it is observed that HLA alleles may play a role in the pathogenesis of coronavirus infections, as in many viral infections. There are also controversial results on the role of single HLA alleles in COVID-19 patients.

## Conclusion

In the present study, in addition to lymphopenia in COVID-19 patients, it was observed that the CD8^+^ T and NK cell levels and also the cytotoxic capacity of NK cells were decreased especially in severe patients. The pro-inflammatory cytokine levels were elevated in severe COVID-19 patients, but it is observed that both Treg and IL-10-expressing CD4^+^ T, CD8^+^ T and NK cells levels were gradually increased from mild to severe patients. These results might suggest that a protective response against the harmful effect of cytokine storm was seen in COVID-19.

## Data availability statement

The original contributions presented in the study are included in the article/**Supplementary Material**. Further inquiries can be directed to the corresponding author.

## Ethics statement

The studies involving human participants were reviewed and approved by Ethics Committee of Istanbul Faculty of Medicine, Istanbul University 11/05/2020-81367. The patients/participants provided their written informed consent to participate in this study.

## Author contributions

Statement of authors contribution: MG, FBO, IT, GD, MK, KK, and VY conceived of the presented idea. FBO, GD, AG, and ET had the approval from the ethical committe. MK, SB, and NS have enrolled the patients and transfered the blood samples. MG, FBO, IT, VY, UK, NA, EC, CC, FSO, KK, and GD performed all experiments and analyzed the data. MG, FBO, IT, VY, MK, AG, ET, SB, and GD supervised findings of this work. OO performed the correlation network analyses. All authors discussed the results and contributed to the final manuscript.

## Funding

This work was supported by Istanbul University BAP and Project number: TSG-2020–36834 and 2019K12-149071.

## Acknowledgments

We thank Abdullah Yilmaz, Gunes Cengiz, and Yasemin Karagoz for excellent technical assistance and data collection; Melek Yanasik for sample transfer, Suzan Cinar and Atahan Cagatay for comments to manuscript. 

## Conflict of interest

The authors declare that the research was conducted in the absence of any commercial or financial relationships that could be construed as a potential conflict of interest.

## Publisher’s note

All claims expressed in this article are solely those of the authors and do not necessarily represent those of their affiliated organizations, or those of the publisher, the editors and the reviewers. Any product that may be evaluated in this article, or claim that may be made by its manufacturer, is not guaranteed or endorsed by the publisher.
