# Supplementary material for: Immune modulation as a consequence of SARS-CoV-2 infection
Source: Front Immunol. 2022 Aug 30;13:954391. doi: 10.3389/fimmu.2022.954391 (PMC9468265; doi:10.3389/fimmu.2022.954391)
Supplement: Supplementary file 1 [file Table_1.docx]

**Supplements**

**Table 1. S**ensitivity of multiplex assay and C3a-C5a proteins

| **Parameter** | **Range** | **Sensitivity* in Serum** |
| --- | --- | --- |
| **IL-1β** | ND – 20.4 pg/mL | 1.5 pg/mL |
| **IFN-α2** | ND – 155.8 pg/mL | 2.1 pg/mL |
| **IFN-β** | ND – 34.6 pg/mL | 1.5 pg/mL |
| **IFN-γ** | ND – 15.0 pg/mL | 1.3 pg/mL |
| **TNF-α** | ND – 27.0 pg/mL | 0.9 pg/mL |
| **IL-6** | ND – 13.1 pg/mL | 1.5 pg/mL |
| **IL-10** | ND – 9.9 pg/mL | 2.0 pg/mL |
| **IL-18** | 53.7 – 276.9 pg/mL | 1.3 pg/mL |
| **GM-CSF** | ND – 4.3 pg/mL | 0.92 pg/mL |
| **C3a** | 0.31 – 20 ng/mL | 0.19 ng/mL |
| **C5a** | 78.13 – 5000 pg/mL | 46.88 pg/mL |

ND: Non Detectable, * minimum detectable concentrations (MDCs)
